# Detection and Classification of Uniform and Concentrated Wall-Thinning Defects Using High-Order Circumferential Guided Waves and Artificial Neural Networks

**DOI:** 10.3390/s23146505

**Published:** 2023-07-18

**Authors:** Donatas Cirtautas, Vykintas Samaitis, Liudas Mažeika, Renaldas Raišutis

**Affiliations:** Prof. K. Baršauskas Ultrasound Research Institute, Kaunas University of Technology Lithuania, Barsausko St. 59, LT-51423 Kaunas, Lithuania

**Keywords:** ultrasonic guided waves, high-order modes, defect classification, corrosion, wall thinning, pipeline structures, artificial neural networks

## Abstract

Pipeline structures are susceptible to corrosion, leading to significant safety, environmental, and economic implications. Existing long range guided wave inspection systems often fail to detect footprints of the concentrated defects, which can lead to leakage. One way to tackle this issue is the utilization of circumferential guided waves that inspect the pipe’s cross section. However, achieving the necessary detection resolution typically necessitates the use of high-order modes hindering the inspection data interpretation. This study presents the implementation of an ultrasonic technique capable of detecting and classifying wall thinning and concentrated defects using high-order guided wave modes. The technique is based on a proposed phase velocity mapping approach, which generates a set of isolated wave modes within a specified phase velocity range. By referencing phase velocity maps obtained from defect-free stages of the pipe, it becomes possible to observe changes resulting from the presence of defects and assign those changes to the specific type of damage using artificial neural networks (ANN). The paper outlines the fundamental principles of the proposed phase velocity mapping technique and the ANN models employed for classification tasks that use synthetic data as an input. The presented results are meticulously verified using samples with artificial defects and appropriate numerical models. Through numerical modeling, experimental verification, and analysis using ANN, the proposed method demonstrates promising outcomes in defect detection and classification, providing a more comprehensive assessment of wall thinning and concentrated defects. The model achieved an average prediction accuracy of 92% for localized defects, 99% for defect-free cases, and 98% for uniform defects.

## 1. Introduction

Pipeline structures play an essential role in the transportation of various fluids, such as oil, gas, and water [1,2,3]. They are critical to the energy, water supply, and petrochemical industries, among others, and ensuring their structural integrity and reliable operation is vital for public safety, environmental protection, and economic stability [4]. Corrosion is a significant factor contributing to the degradation of pipelines and is responsible for approximately up to 25% of all pipeline incidents. It can lead to various issues such as leaks, ruptures, and in extreme cases, even catastrophic failures [5]. For instance, corrosion has been identified as a primary factor in major pipeline incidents, such as the Kalamazoo River pipeline leak in the United States. This incident led to the release of 20,000 barrels of oil and required cleanup operations with a cost exceeding USD 700 million [6]. A comprehensive study conducted between 2002 and 2013 [7] has identified the primary factors responsible for pipe-related incidents, accounting for over 75% of the recorded cases. These factors include third-party interference, external corrosion, material failure, and internal corrosion. Each of these factors poses a significant risk to pipeline integrity and underscores the importance of timely detection and identification of defects, particularly localized corrosion [8,9].

Numerous non-destructive testing (NDT) techniques have been employed for in-line pipeline inspection, such as magnetic flux leakage, radiography, and ultrasonic testing [10,11,12,13]. Ultrasonic guided waves (UGW) have gained significant attention in transition from traditional ultrasonic testing due to their ability to inspect long-range distances and sensitivity to various defect types, including corrosion, cracks, and weld defects [14,15]. UGW provides qualitative measurements of wall loss defects and is complemented by conventional NDT methods for assessing details of suspected areas. Different modes in guided waves offer unique characteristics for detecting specific defect types or providing complementary information for defect identification in cylindrical structures such as pipelines [16].

Existing guided wave methods often encounter challenges in detecting localized defects, as they typically yield weak signatures in recorded signals, resulting in concealed defect responses. There is a trade-off between the inspection distance and resolution in guided wave systems. Conventional systems that utilize fundamental modes at relatively low frequencies provide long propagation distances but are less sensitive to localized and pitting defects [17,18].

Novel techniques have emerged that utilize thickness loss measurements around the circumference of pipes as a tool for more accurate and relatively large-scale assessment of corrosion [19,20]. These techniques frequently utilize high-order guided wave modes that propagate along the circumference of the pipe and are sensitive to changes in wall thickness [21,22]. When these techniques are combined with specialized scanners, they can be used as a rapid pipe cross section screening method, allowing for the real-time generation of wall thickness maps. As a result, potentially suspicious areas can undergo further evaluation using conventional non-destructive testing (NDT) methods.

Pipe cross-section inspections offer the advantage of shorter propagation distances, allowing for the utilization of higher frequency waves with shorter wavelengths. Although these waves are traditionally prone to significant attenuation, especially when subjected to liquid loading, they can still be effectively used at distances of up to a few meters, which is generally suitable for most common pipe diameters. As a result, there has been significant research focused on exploring the use of high-order guided wave modes for detecting wall thinning and corrosion around the circumference of the pipe. Notable studies in circumferential guided wave inspection have been conducted by P. Khalili and F. Cegla, who analyzed SH1 and A1 modes for wall thinning detection [23,24,25]. While high-order modes have been studied, there is still a wide range of modes and their interaction with various types of defects that can be further explored. Most current methods also lack automation and rely on single signal analysis for defect detection [16,26].

As screening methods for pipe inspection often generate large amounts of data, there is a risk of missing defect signatures. To improve defect detection capabilities, various artificial intelligence (AI) methods have been explored. Recent studies have utilized deep learning techniques, including convolutional neural networks (CNN) [27,28] and deep GFresNet [29], to enhance the accuracy of defect detection and classification in pipeline or plate structures, achieving high accuracy rates of up to 97% [27,28,29,30]. Ultrasonic guided waves (UGW) have also shown promising results in non-invasive inspection, particularly in detecting gas diversions in high-density polyethylene (HDPE) pipes using deep neural networks (DNNs), with defect classification accuracy of up to 99.6% [31]. In recent investigations, a CNN was trained on low-order guided waves propagating along a hollow cylindrical shell, resulting in remarkable defect detection performance. Specifically, in buried pipes, the CNN achieved a 100% detection rate in soil and 97.3% in concrete, at a signal-to-noise ratio (SNR) of 100 dB, while 83.3% and 42% was achieved at SNR of 70 dB [32]. Furthermore, another research study utilized electromagnetic acoustic transducers (EMAT) to generate low-order guided waves in plates, employing machine learning (ML) techniques. The analysis demonstrated the potential of ML in accurately determining defect size, achieving an impressive accuracy of 97.06% through full mode separation [33]. These advancements highlight the potential of combining machine learning and deep learning techniques with guided waves to improve the accuracy, speed, and automation of defect detection and classification [32,33,34,35,36]. However, current inspections primarily rely on fundamental modes for training neural networks, mainly due to the ease of exciting and analyzing these modes. Shear modes in particular are commonly employed with EMAT transducers. Conversely, high-order modes have the potential to offer improved resolution and unique characteristics that can provide deeper insights into existing damage. Nevertheless, their current usage is limited due to the challenges posed by multiple co-existing modes, complex dispersive characteristics, overlapping in the time domain, and intricate analysis.

In our previous paper [37], we demonstrated the suitability of high-order modes, such as S3 and A1, for detecting wall-thinning defects and means for their isolated excitation. In this paper, we combine the findings of our previous work on the ultrasonic method for AI-assisted corrosion detection and classification. The method is based on inspecting the cross-section of the pipe and utilizes two phased arrays arranged in a pitch–catch configuration, along with high-order guided waves (S1, S2, S3 and A1) that propagate around the pipe’s circumference. This paper proposes a new approach for analyzing the propagation of high-order modes around the circumference of the pipe, resulting in a propagation time vs. phase velocity plot which we have called the phase velocity mapping technique. To the best of our knowledge, no similar approach has been previously employed for the analysis of the propagation of multiple high-order guided wave modes or utilizedfor the purpose of corrosion detection. The proposed phase velocity maps are generated by selectively exciting different high-order guided wave modes within a specific phase velocity range and combining the received responses into a single plot that encompasses information regarding the amplitude, phase, and group velocity of guided waves. The generated phase velocity map contains various modes that can be generated in the structure, enabling observation of emerging changes resulting from the presence of defects. Since each mode exhibits unique through-thickness displacements, this method can ensure sensitivity to a broader range of defects. Moreover, by incorporating multiple high-order guided wave modes and operating in the high-frequency range, we can enhance sensitivity to defects and gain a comprehensive understanding of corrosion damage as a combination of different mode behaviors. While the phase velocity maps primarily depictthe phase velocity of guided waves, it is important to note that the plot also retains information about the group velocity, as it includes a time axis. The generated phase velocity maps were used to train a VGG-16 convolutional neural network for defect classification. Three classes were considered: no defect, localized defect, and uniform or distributed defect. The neural network was trained with synthetic data, utilizing FEM models that represented different stages of damage, considering various defect positions and wall thinning. The FEM models were specially tuned to closely match the experimental data. A total of 412 cases were simulated and fed to the neural network. To assess the network’s ability to predict the class of defects, the model was tested with synthetic and experimental data from a mock-up with artificial defects. The model demonstrated an average prediction accuracy of 92% for localized defects, 99% accuracy for no defect cases, and 98% for uniform defects. In this paper, we outline the fundamental principles behind the proposed phase velocity mapping technique and the artificial neural network models employed for classification tasks. We provide a detailed description of the numerical models used for simulating defected cases and discuss the verification results obtained from actual mock-up data. The results demonstrate that phase velocity maps can offer reliable data for neural networks to accurately predict the defect class. Although the training was conducted using synthetic data, it opens up opportunities to enhance the prediction capabilities of the models by incorporating new cases in the future.

## 2. Object under Investigation

**Figure 1:**
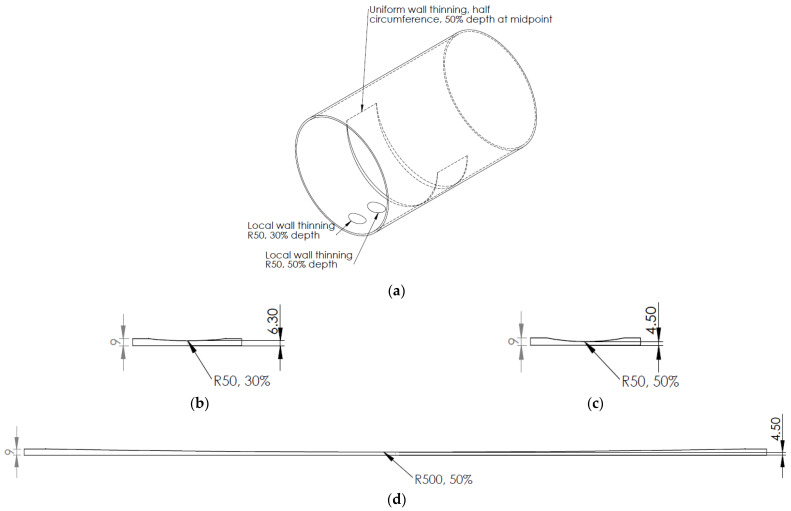
A 3D view of the pipe segment (**a**); cross-sectional images of the defects: localized corrosion with a depth of 30% (**b**) and 50% (**c**) of initial wall thickness; and uniform corrosion with a thickness loss reaching 50% of the initial wall thickness at the midpoint (**d**).

In this paper, we investigate a pipe segment made of Steel Alloy 1020, with an outer diameter of 636.6 mm, a circumference of 2 m, and a wall thickness of 9 mm. The following material properties were considered to describe the material: a density of 7850 kg/m^3^, Young’s modulus of 207 GPa, and Poisson’s ratio of 0.3. The mock-up consisted of two types of artificial defects that simulate wall thickness loss due to corrosion, namely, localized and uniform wall thinning. Localized corrosion was represented by a cylindrical cut with a radius of 50 mm and a depth of 30% and 50% of the initial pipe wall thickness (reference as localized 30% and localized 50%). The uniform defect was implemented as a 1000 mm zone around the circumference of the pipe with gradual wall thickness loss, reaching 50% of the initial wall thickness at the midpoint (referenced as uniform 50%). The defect-free zone for the reference measurement was also designated on the pipe mock-up. Figure 1 presents a 3D view of the pipe segment and cross-sectional images of the considered defects.

**Figure 2:**
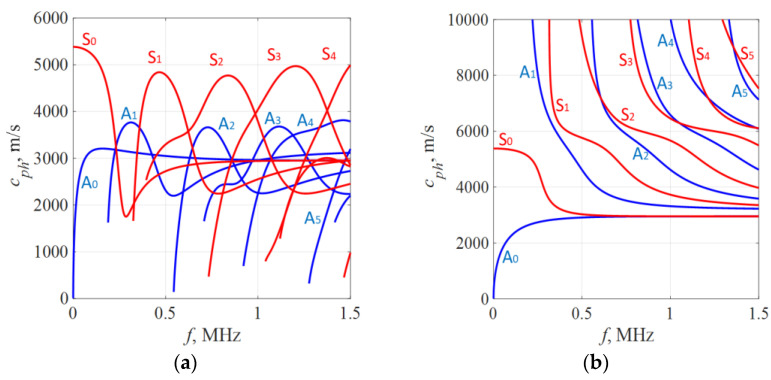
Dispersion curves in a 9 mm SteelAlloy1020 plate: (**a**) group velocity; (**b**) phase velocity.

**Figure 3:**
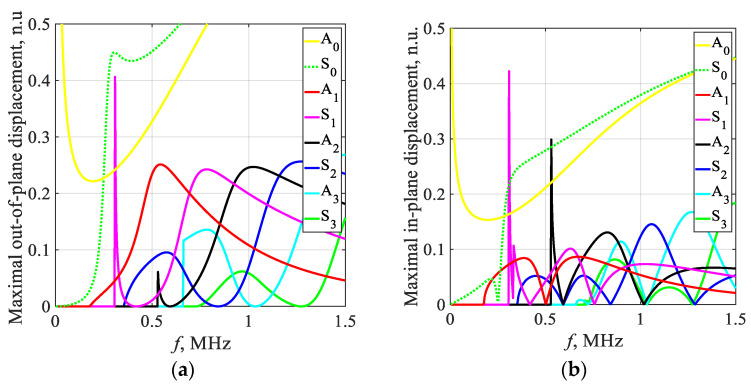
Maximal displacement vs. frequency: (**a**) out-of-plane; (**b**) in-plane.

The dispersion curves for phase and group velocity, as well as the corresponding in-plane and out-of-plane displacements at the surface of the structure, were calculated using a semi-analytical finite element technique (SAFE). In this analysis, a 1D SAFE mode was employed, considering the unrolled SteelAlloy1020 pipe with the specified material properties. Previous studies have suggested that the Rayleigh–Lamb dispersion equations for plates can be used as an approximation to obtain dispersion relations in the circumferential direction of thin cylindrical shells [20,38]. This approximation holds true for thin-walled pipes where the ratio between the inner (r) and outer (R) radius is r/R > 0.95. In our study, we consider a structure with r/R > 0.97. To calculate the dispersion curves, the cross-section of the 9 mm plate was discretized into 20 elements, each with a size of 0.45 mm and composed of 3 nodes. The resulting estimated dispersion curves are presented in Figure 2. Our focus in this paper is on working with a 1 MHz excitation frequency, where various modes exist, offering different opportunities for damage detection. As explored in our previous paper, at this specific frequency, the S3 and A1 modes have shown high potential for assessing pipeline corrosion. In addition to these modes, other co-existing modes such as S1 and S2 will also be exploited in this study. The in-plane and out-of-plane displacements at the surface of the considered structure, which are depicted in Figure 3, play a crucial role in determining the excitability and detectability of the modes, as well as their energy losses to the surrounding media.

## 3. Phase Velocity Mapping Method for the Assessment of Emerging Pipeline Defects

The proposed method for detecting and classifying corrosion-type defects is based on the isolated excitation of different guided wave modes within a specific phase velocity range and the arrangement of the received responses into a propagation time vs. phase velocity plot, i.e., a so-called phase velocity map. Such a map can be used to explore the waves propagating in defect-free and defected regions of the pipe, while the changes in each mode can be attributed to the specific defect. The generated phase velocity maps can then be utilized to train neural networks for AI-assisted defect detection and classification. In the following paragraphs, we will provide a brief explanation of the fundamental principles underlying the phase velocity mapping method.

It is known that an isolated guided wave mode can be generated using a phased array with a fixed elementary pitch by applying a delay in the emission to excite the desired phase velocity [39]. This delay creates an angled plane wave front that is associated with the longitudinal velocity of the waveguide and the phase velocity.

Specifically, a time delay of $t_{st_{i}}$ needs to be applied to each element i of the array to achieve the propagation of the plane wave at the desired angle, which is associated with the phase velocity of the mode at the specific excitation frequency. If the phased array is coupled to the object with a wedge, the delay time for individual array elements can be expressed as follows:

(1) $$t_{st_{i}}=L_{pitch}\cdot \frac{i-1}{c_{ph}\left(f_{c}\right)-\frac{c_{w}}{\sin\left(\alpha_{prism}\right)}},$$

where i is the element number; $L_{pitch}$ is the distance between array elements (pitch); $c_{ph}\left(f_{c}\right)$ is phase velocity at center excitation frequency $f_{c}$; $c_{w}$ is wave velocity in wedge; and $\alpha_{prism}$ is the wedge angle.

At the reception side, each array element records individual signals from the wave fired at a specific angle, which are then summed to generate an output signal:

(2) $$u_{\Sigma }\left(t\right)=\sum_{i=1}^{N}u_{i}\left(t-t_{st,i}\right),$$

where $u_{i}\left(t\right)$ represents the signal measured by *i*th phased array element; N is the total number of elements in phased array; and $t_{st,i}$ is the phasing delay calculated according to Equation (1). To construct a phase velocity map of the structure, we can employ a set of phased array delay laws during the excitation process. This enables the generation of modes at different angles and, consequently, at different phase velocities. On the reception side, the signals corresponding to each specific propagation angle can be combined into a single signal. By collecting such signals at different excitation angles and correlating the excitation angles with specific phase velocities, we can generate a plot that shows the relationship between propagation time and phase velocity Equation (3), which ultimately forms a phase velocity map. The phase velocity map Equation (4) can be mathematically expressed as follows:

(3) $$u_{\Sigma }\left(t,\;c_{ph}\left(f_{c}\right)\right)=\sum_{i=1}^{N}u_{i}\left(t-L_{pitch}\;\cdot \frac{i-1}{c_{ph}\left(f_{c}\right)-\frac{c_{w}}{\sin\left(\alpha_{prism}\right)}}\right)$$

(4) $$U_{\Sigma }=\{u_{\Sigma }\left(t,\;c_{ph_{k_{j}}}\left(f_{c}\right)\right),u_{\Sigma }\left(t,\;c_{ph_{k_{j}+1}}\left(f_{c}\right)\right),\ldots ,u_{\Sigma }\left(t,\;c_{ph_{k_{n}}}\left(f_{c}\right)\right)\},$$

where $u_{\Sigma }\left(t,c_{ph}\left(f_{c}\right)\right)$ represents waveform at specific phase velocity $c_{ph}\left(f_{c}\right)$; t is the time variable; *u_i_* is the signal measured by the *i*th phased array element; k defines the range of phase velocity values from the initial $k_{j}$ to $k_{n}$, $U_{\Sigma }$ defines phase velocity map.

**Figure 4:**
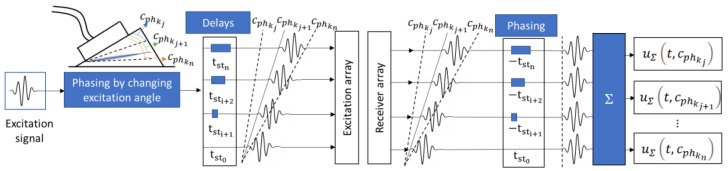
The concept of the phase velocity mapping technique.

**Figure 5:**
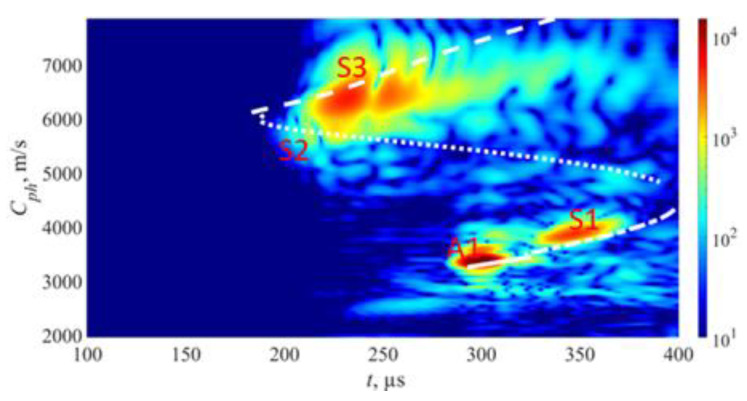
An example of the phase velocity map captured on a defect-free steel pipe with a wall thickness of 9 mm with theoretical dispersion curves where colormap represents measured and further processed amplitude by phased array elements in arbitrary units.

The concept of the phase velocity mapping technique is illustrated in Figure 4. An example of the phase velocity map captured on a defect-free steel pipe with a wall thickness of 9 mm is presented in Figure 5. To generate this plot, two 1-MHz 32-element arrays were arranged in a pitch–catch configuration with distance of 715 mm between plexiglass wedges. Excitation was applied considering a phase velocity range of 1960 m/s to 7860 m/s. The phase velocity map demonstrates the generation of multiple modes at different phase velocities, allowing for the exploration of the modes present in the structure. As defects appear, changes in the phase velocity map will emerge, which can be utilized for defect detection and training of classification models. It is important to note that the phase velocity map and the number of co-existing modes are influenced by the excitation frequency, bandwidth, and dispersive properties of the material. Additionally, the aperture of the array determines the mode purity in the recorded signals. In this example, we only included the direct propagating modes and limited the reconstruction time domain to 400 μs.

## 4. Experimental Validation

In this section, we report our exercising of the phase velocity mapping method on the experimental sample—an industry-relevant pipe section described in Section 2.

**Figure 6:**
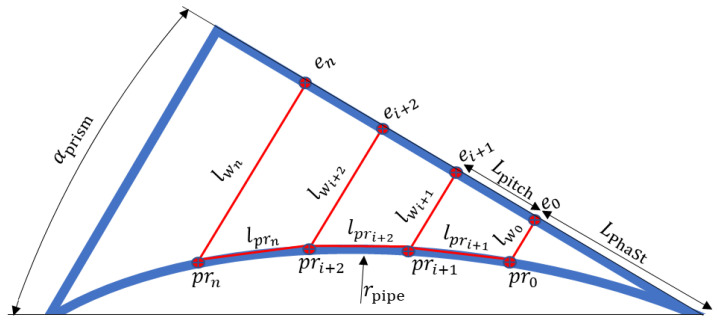
Plexiglass concave wedge and phased array wave propagation projections.

To estimate time delays when implementing a concave wedge, as shown in Figure 6, it is necessary to establish a wavefront that no longer remains flat but instead resembles the curvature of the pipe. This requires the use of several linear equations. Firstly, the center coordinates of each array element *e* need to be determined, taking into account the actual array position on the wedge. In other words, the distance *L*_PhaSt_ between the first array element and the corner of the wedge must be known. Then, projection points *pr* for each array element onto the surface of the pipe must be determined, considering the propagation from the array element center to the pipe’s surface. Finally, with the establishment of propagation distances, time delays can be evaluated for each array element, thus forming a concave wavefront.

The *x* $e_{i_{x}}$ and the *y* $e_{i_{y}}$ coordinates of the *i*th element of the phased array positioned on an angled wedge can be computed using the following formulae:

(5) $$\begin{array}{c} e_{i_{x}}=\left(\left(n-1\right)\cdot L_{pitch}\right)\cdot cos\left(180-\alpha_{prism}+\sin^{-1}\left(\frac{\left(\left(n-1\right)\cdot L_{pitch}\right)+L_{PhaSt}}{cos\left(\alpha_{prism}\right)\cdot 2\cdot r_{pipe}}\right)\right)+\\ 10\cdot sin\left(180-\alpha_{prism}+\sin^{-1}\left(\frac{\left(\left(N-1\right)\cdot L_{pitch}\right)+L_{PhaSt}}{cos\left(\alpha_{prism}\right)\cdot 2\cdot r_{pipe}}\right)\right) \end{array}$$

(6) $$\begin{array}{c} e_{i_{y}}=\left(\left(n-1\right)\cdot L_{pitch}\right)\cdot sin\left(180-\alpha_{prism}+{sin}^{-1}\left(\frac{\left(\left(n-1\right)\cdot L_{pitch}\right)+L_{PhaSt}}{cos\left(\alpha_{prism}\right)\cdot 2\cdot r_{pipe}}\right)\right)-\\ 10\cdot cos\left(180-\alpha_{prism}+{sin}^{-1}\left(\frac{\left(\left(N-1\right)\cdot L_{pitch}\right)+L_{PhaSt}}{cos\left(\alpha_{prism}\right)\cdot 2\cdot r_{pipe}}\right)\right)+r_{pipe} \end{array}$$

where $L_{PhaSt}$ determines the distance from wedge corner till first element center of the phased array; $r_{pipe}$ is pipe radius; and n is the element number. In this case, *n* ranges from 1 to *N*.

The coordinates of the projection points to the pipe surface can be estimated according to the following:

(7) $${pr}_{i_{x}}=\frac{-2\cdot a\cdot b+\sqrt{4\cdot a^{2}\cdot b^{2}-4\cdot \left(1+a^{2}\right)\cdot \left(b^{2}-{r_{pipe}}^{2}\right)}}{2\cdot \left(1+a^{2}\right)}$$

(8) $${pr}_{i_{y}}=a\cdot {pr}_{i_{x}}+b$$

(9) $$a=-\frac{e_{N_{x}}-e_{0_{x}}}{e_{N_{y}}-e_{0_{y}}}$$

(10) $$b=\frac{e_{i_{y}}+\left(e_{i_{x}}\cdot (e_{0_{x}}-e_{N_{x}})\right)}{e_{0_{y}}-e_{N_{y}}}.$$

where the *x*-coordinate of the projection point for a specific element ${pr}_{i_{x}}$ is determined by Equation (7). It involves solving a quadratic equation using the coefficients a and b. The *y*-coordinate of the projection point for the same element ${pr}_{i_{y}}$ is calculated using Equation (8). The coefficient a is derived from Equation (9), which represents the slope of the line perpendicular to the phased array surface. It is determined by taking the difference between the *x*-coordinates of the last and first elements ($e_{N_{x}}$ and $e_{0_{x}}$) and dividing it by the difference in *y*-coordinates ($e_{N_{y}}$ − $e_{0_{y}}$). The coefficient b is calculated using Equation (10), which represents the intercept of the line perpendicular to the phased array surface where $e_{i_{x}}$ and $e_{i_{y}}$ is current element *x* and *y* coordinates. In summary, a and b are the coefficients representing the line slopes of the projections perpendicular to the phased array surface for each element.

The projection length of the wave through the wedge $l_{w_{i}}$ is calculated:

(11) $$l_{w_{i}}=\sqrt{{\left({pr}_{i_{x}}-e_{i_{x}}\right)}^{2}+{\left(\;{pr}_{i_{y}}-e_{i_{x}}\right)}^{2}}.$$

Additionally, the projection distance $l_{{pr}_{i+1}}$ for each sequential pair of elements, where time of flight is considered as phase velocity, is computed:

(12) $$l_{{pr}_{i+1}}=\sqrt{{\left({pr}_{i+1_{x}}-{pr}_{i_{x}}\right)}^{2}+{\left(\;{pr}_{i+1_{y}}-{pr}_{i_{y}}\right)}^{2}}.$$

Equation (13) is then employed to determine time delays for each element e to propagate a wavefront through the wedge onto the plate surface, exciting guided waves at the desired phase velocity:

(13) $$t_{{stpr}_{i}}=t_{gw_{i}}-\frac{l_{w_{i}}}{c_{w}}$$

(14) $$t_{gw_{i}}=\left\{\begin{array}{c} \sum_{i=2}^{N}\left(\frac{l_{{pr}_{i}}}{c_{ph\left(f_{c}\right)}}\right)\;,\;i>1\\ 0,\;i=1 \end{array}\right.,$$

where $l_{w_{i}}$ corresponds to the distance between projection on the pipe surface from a specific array element located at the surface of the wedge; and $c_{w}$ is ultrasound velocity in plexiglass wedge. It represents the distance travelled by the wave in the wedge and indicates the time required for the wave to propagate the wedge and reach the pipe surface. $t_{gw_{i}}$ calculates the guided wave time of flight from the first projection to the desired element’s delay projection. These calculations in Equation (14) mean that $t_{gw_{i}}$ for the initial element is equal 0 and equal to $\sum_{i=2}^{N}\left(\frac{l_{{pr}_{i}}}{c_{ph\left(f_{c}\right)}}\right)$ for the subsequent array elements.

The outcome of these calculations provides the time delay necessary for each phased array element to propagate a wave front through the concave wedge and onto the pipe surface, allowing for the generation of guided waves at the desired phase velocity.

**Figure 7:**
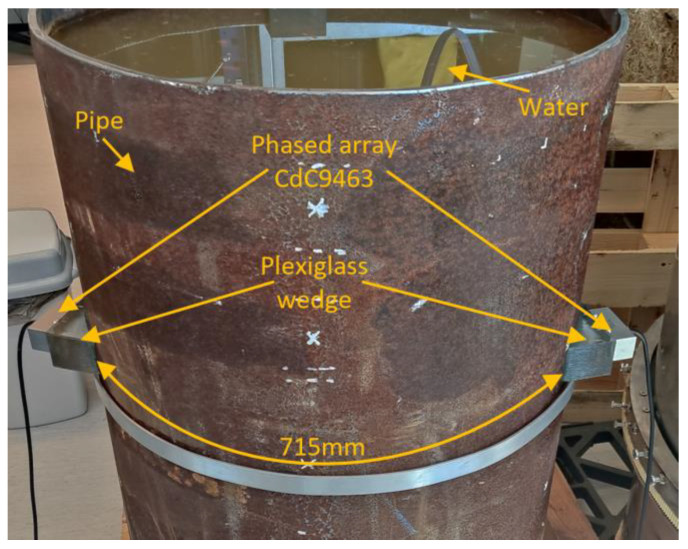
Experimental setup for assessment of phase velocity mapping method.

**Figure 8:**
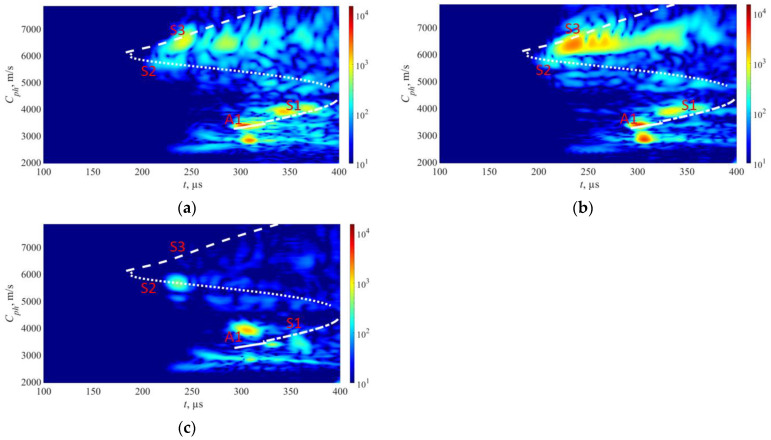
Experimental results from pipe mock-up: (**a**) localized defect 30% of pipe wall thickness; (**b**) localized defect 50% of pipe wall thickness; (**c**) uniform defect 50% of pipe wall thickness. The overlaid white lines represent theoretical dispersion curves. The color bars are represented in arbitrary units and scaled based on the magnitude of the S3 mode in the defect-free region.

The approach described above was utilized in experiments to generate phase velocity maps on a pipe with localized and uniform defects, as detailed in Section 2. The detailed drawing of the pipe mock-up was presented in Figure 1. Two 1-MHz 32-element Imasonic CdC9463-2 phased arrays were employed for this purpose, with a pitch of 2.05 mm and an active aperture of 65.1 mm. These arrays were arranged in a pitch–catch configuration and positioned 715 mm apart around the circumference of the pipe using lithium grease as a couplant, as illustrated in Figure 7. To account for the pipe’s curvature, each array was screwed on a concave 33° plexiglass wedge. The same lithium grease was used to couple the phased array to the wedge. To ensure the proper attachment force, a cargo belt was used. To generate the required plane waves, delays were applied to the array elements following Equations (13) and (14). The delay laws were calculated for velocities ranging from 1960 m/s to 7860 m/s, with a step size of 50 m/s, taking into consideration the angle of the wedge. To replicate in situ conditions, the pipe segment was filled with water. The multichannel data acquisition system Dasel Sitau (Dasel Sistemas, Madrid, Spain) with 128 parallel channels was employed to generate and record signals of propagating modes. The phase velocity map images generated at defect-free and defected locations of the pipe are presented in Figure 5 and Figure 8. The color scales of the experimental phase velocity maps are expressed in arbitrary units and scaled according to the magnitude of the S3 mode at the defect-free location.

In Figure 5, the results of the inspection at a defect-free location are presented. The S3, A1, and S1 wave modes are clearly visible, each exhibiting distinct and identifiable phase velocity ranges. The weak fingerprint of the S2 mode can also be observed. In our previous paper [37], it was demonstrated that both S3 and A1 modes at *f* × *d* of 9 MHz × mm can be effectively used for detecting uniform and concentrated wall-thinning defects as these modes have relatively short wavelengths, low attenuation, and can be employed for inspecting water-filled pipes using conventional ultrasonic phased arrays. In this case, we also took into account other co-existing modes such as S1 and S2, as they are expected to offer supplementary information for defect detection and classification. However, it is important to note that the S1 mode has higher leakage losses compared to the S3 and A1 modes. Therefore, increasing the inspection distance may result in the absence of the S1 mode.

Moving on to Figure 8a, it shows the outcomes of inspecting a sample containing a localized defect at a depth of 30%. Here, all wave modes—S3, S2, S1, and A1—demonstrate reduced responses in contrast to defect-free state. The S2 mode is nearly undetectable. Despite this overall weakening, the S3, A1, and S1 modes remain visible. In contrast, Figure 8b demonstrates the presence of a localized defect at a depth of 50%. In this scenario, the S3 wave modes exhibit less attenuation compared to the case of a localized defect with a depth of 30%. This can be explained by analyzing the out-of-plane displacements of the S3 mode at the outer surface of the pipe. According to the out-of-plane displacements (Figure 3a), an increase in defect depth should result in weakened displacements of the S3 mode at the outer surface. However, since the defect is highly localized, a sudden loss of wall thickness leads to only minor changes in the out-of-plane displacement for the S3 mode. The main influencing factor in this case is the surface roughness of the pipe. At a frequency–thickness product of approximately 9 MHz × mm, the out-of-plane displacements of the S3 mode exhibit a local maximum, indicating that the surface roughness will result in high attenuation of the S3 mode. As the frequency decreases, the out-of-plane displacement of the S3 mode decreases, resulting in less attenuation caused by surface roughness. Consequently, defects with greater depths within a certain range will show less attenuation of the S3 mode. Additionally, in both cases of localized defects, a noticeable change in the arrival time of the S1 mode can be observed. It can be observed that as the wall thickness loss of the localized defect increases, the propagation velocity of the S1 mode also increases.

During the analysis of a sample exhibiting uniform wall thinning with a depth of 50% at the mid-point, as shown in Figure 8c, it is observed that the S3 wave mode is noticeably absent. This can be attributed to the change in wall thickness of the pipe, resulting in a different *f* × *d* product that is no longer 9 MHz × mm. Consequently, the *f* × *d* product aligns with the cut-off frequency of the S3 mode (Figure 2).

The phase velocity of the S1 mode undergoes significant changes when a uniform defect is present, causing the S1 mode to arrive earlier due to the thinning effect. Additionally, the A1 wave mode experiences significant attenuation, leading to reduced noise levels. Interestingly, the absence of the S3 mode in scenarios of uniform thinning suggests a different type of defect compared to localized defect cases, where the S3 mode is clearly visible. This finding supports the theory that the existing cut-off frequency of the S3 mode prevents its propagation in a uniformly thinned structure. Therefore, it can serve as an indicator of uniform defects and help identify cases where the wall thickness loss is approximately 50%.

Furthermore, the arrival times of the S1 and A1 modes serve as useful indicators for estimating the thickness and extent of the uniform defect. These observations are further supported by the dispersion curves, which indicate that as the sample thickness decreases, the group velocity of the A1 wave mode decreases while that of the S1 wave mode increases.

Overall, the figures presented above illustrate the complex interaction of various wave modes under different defect conditions, providing insights into the behavior and detection of these modes at varying levels of damage. It is important to note that in this case, only directly transmitted modes are considered.

## 5. Classification of Defects Using Neural Networks

### 5.1. Description of the Neural Network Architecture

**Figure 9:**
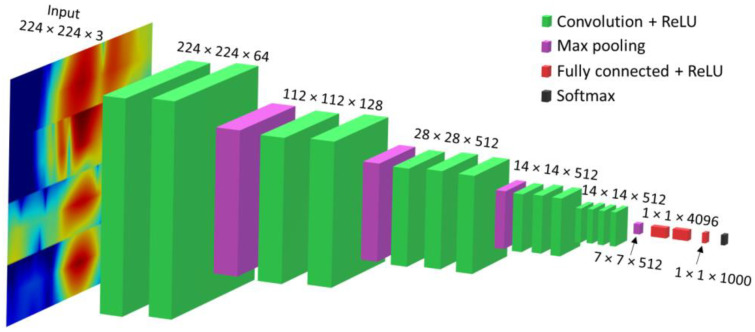
VGG-16 neural network architecture.

The VGG-16 (Visual Geometry Group-16) neural network was used in this paper to classify the corrosion type defects based on estimated phase velocity maps. The VGG-16 is a convolutional neural network architecture that was developed by the Visual Geometry Group at the University of Oxford [40]. The VGG-16 network consists of 16 layers, including 13 convolutional layers, 5 max-pooling layers, and 3 fully connected layers. One of the key features of the VGG-16 network is that it uses a very small kernel size (3 × 3) for all convolutional layers. This allows for more layers to be stacked while maintaining a manageable number of parameters, which helps to improve the accuracy of the network. Another important aspect of the VGG-16 network is that it uses a large number of filters in each convolutional layer, which helps to capture a wide range of features at different levels of abstraction. This, along with the small kernel size, contributes to the network’s ability to extract rich and detailed features from the input image. The architecture of the selected VGG-16 neural network is presented in Figure 9.

The VGG-16 neural network architecture is similar to that of VGG-19, ResNet-50, Inception-V3, and DenseNet-121. However, the simplicity of VGG-16 and its ability to handle low-resolution images make it a preferred choice over the others. Therefore, the selection of VGG-16 was motivated by its efficiency in handling image data with limited computational resources [40].

### 5.2. Description of Synthetic Model for Training of VGG-16 Neural Network

To train the selected neural network effectively, a sufficient amount of training data must be generated. However, since the experimental mock-up contained only a limited number of defect cases, it was decided to train the neural network using synthetic phase velocity maps. To accomplish this, a series of finite element models were created to simulate phase velocity maps with different defect types, positions, and wall thinning extents. Although training with synthetic data can present challenges, such as ensuring that the digital model corresponds to the responses generated on the actual mock-up, it was determined to be a viable option. To ensure that the model accurately reflects reality, adjustments were made to the model to mimic the results from the experimental measurements. A 2D model was selected for this purpose as it balances modeling time and validity, although diffraction was neglected.

**Figure 10:**
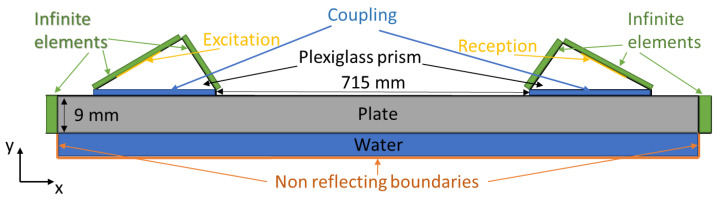
FEM model of the unwrapped pipe.

The configuration of the FEM model is depicted in Figure 10. To simulate the inspection of the pipe cross section, the unrolled pipe technique was employed. In the modeling of wave phenomena in a large diameter, thin-walled pipes often rely on simplified theory, considering the pipe as an unwrapped isotropic plate and recognizing that scattered circumferential modal amplitudes from defects in a pipe are related to guided wave scattering from defects modeled in plates. The validity of this technique has been demonstrated in our previous article and by other authors [19,37,41,42]. As shown in Figure 10, the pipe was as a 9 mm thick steel plate loaded with water from the inner surface as it simulates a pipe in fully operational condition. The unrolled pipe was created as isotropic material using the CPE4R element type and had same material properties as those presented in Section 2. To prevent wave reflection from the edges, CINPE4-type infinite elements were placed around both ends of the steel plate (Figure 10).

The pitch–catch setup of the phased arrays was simulated using a configuration consisting of nodes placed on plexiglass wedges. These nodes were spaced at a distance equal to the pitch of the Imasonic CdC9463-2 phased array utilized in the experiments, which was 2.05 mm. To accurately model the experimental setup, the plexiglass wedges were positioned at a distance of 715 mm from each other. The plexiglass wedges were simulated using CPE3 finite elements, while absorbing boundaries were placed at the edges that were not in contact with the steel. To further replicate the experimental conditions, a water layer was introduced as a couplant between the plexiglass wedges and the plate. The water layer was characterized by a density of 1000 kg/m^3^ and a bulk modulus of 2.2 GPa. The plexiglass material properties were defined as follows: density of 1190 kg/m^3^, Young’s modulus of 5.475 GPa, and a Poisson’s ratio of 0.3569.

A normal excitation force was applied to the surface of the wedges to generate plane waves at different angles. In order to obtain phase velocity maps, the plane waves were calculated for a range of phase velocities spanning from 1960 m/s to 7860 m/s, with an increment of 250 m/s.

**Figure 11:**
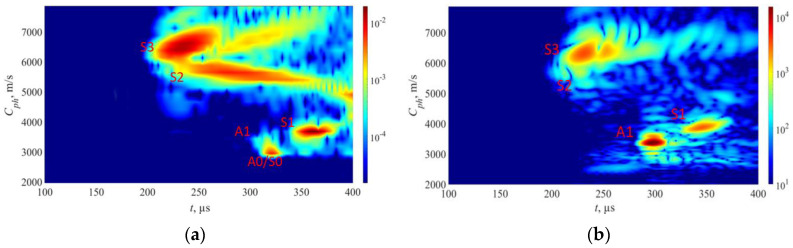
Results from samples without any surface defects: (**a**) FEM results from 9 mm thickness sample; (**b**) experimental results from 9 mm wall thickness pipe sample. The color bars are represented in arbitrary units and scaled based on the magnitude of the S3 mode in the defect-free region.

To verify the accuracy of the synthetic model in relation to the experimental data, it was assumed that a comparison between the experimentally and synthetically generated phase velocity maps would be adequate. It is important to note that the 2D model incorporates certain approximations, and since VGG16 employs images as input data, the comparison of the synthetic and experimental phase velocity maps is considered a pragmatic mean of assessing similarity. The comparison of simulated and experimental phase velocity maps at the defect-free location is presented in Figure 11. Although the magnitude scales in the experimental and FEM images presented in Figure 11 differ, the color scales of both the FEM and experimental results were adjusted based on the magnitude of the S3 mode in the defect-free case. This adjustment ensures that both datasets maximize the magnitude of the S3 mode, which is the main mode of interest in this study. For the case of defects, we maintain the same color scale as in the defect-free case, enabling us to track relative changes in the amplitudes.

Initially, a comparison was made between the experimental and synthetic data at a defect-free location. To assess the similarity of the two phase velocity maps, metrics such as the structural similarity index (SSIM) and the 2D correlation coefficient were used [43]. The SSIM index is based on a multiplicative combination of image luminance, contrast, and structural terms, taking into account local means, standard deviations, and cross-covariance for the image. The 2D correlation coefficient provides a single measure of similarity between two images. The obtained SSIM and 2D correlation coefficients for the FEM simulated and experimental phase velocity maps in the defect-free region of the pipe are 72% and 80%, respectively. Although the absolute values on the color scale differed between the FEM and experimental images, the adjustment of color intensity based on the S3 mode enabled accurate comparison and similarity assessment between the images.

**Figure 12:**
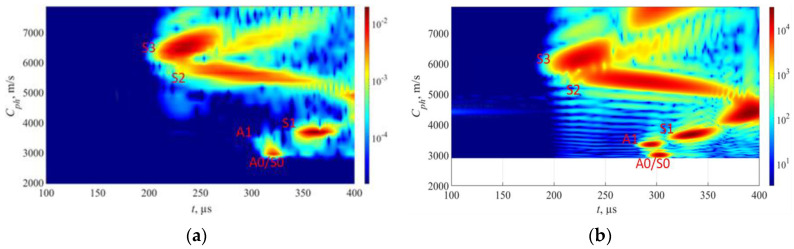
Results from samples without surface roughness: (**a**) FEM results from 9 mm thickness sample; (**b**) experimental results from 10 mm thickness sample with rust free surface. The color bars are represented in arbitrary units and scaled based on the magnitude of the S3 mode in the defect-free region.

The initial FEM results (Figure 12a) revealed the presence of certain wave modes that were not observed in the experimental data (Figure 12b). In this case, strong S2, S0, and A0 wave modes are present in FEM results. One possible explanation for this discrepancy was the presence of surface rust on the experimental pipe, which could have scattered the modes exhibiting high near-surface displacements. To investigate this hypothesis, a steel plate with a rust-free surface and a thickness of 10 mm was employed to identify the propagating modes. Given the 10 mm thickness of the steel plate used in this experiment, the excitation frequency was reduced to 0.93 MHz in order to maintain the same f×d configuration as on the experimental pipe. The rest of experimental parameters were the same as used for the pipe mock-up and FEM model. The experimental results from the clean plate (Figure 10b) showed a strong similarity to the FEM simulation data (Figure 12a), which had assumed a clean surface. This outcome supports the notion that surface rust may have indeed influenced the experimental results.

**Figure 13:**
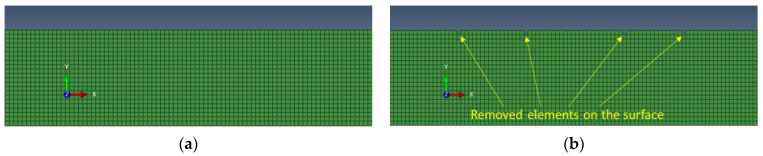
FEM model replication of surface irregularities: (**a**) initial FEM model surface with pure surface; (**b**) updated FEM model with removed elements to mimic affected surface.

In order to better replicate the real pipe experimental data, the FEM model was adapted to account for the irregularities on the pipe surface. This was achieved by randomly removing surface elements in 2–7 mm steps, as shown in the Figure 13. By introducing these surface variations, the FEM simulation aimed to resemble the actual experimental conditions more closely, accounting for factors such as surface rust or other surface imperfections.

**Figure 14:**
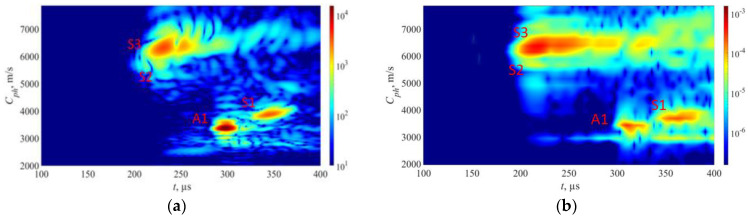
FEM surface adaptation to fit experimental results: (**a**) results from pipe mock-up; (**b**) FEM model that accounts the surface roughness. The color bars are represented in arbitrary units and scaled based on the magnitude of the S3 mode in the defect-free region.

The comparison of experimental data from the pipe mock-up and FEM data, considering the surface roughness, is shown in Figure 14. In Figure 14a, the experimental sample results show the visibility of the S1, S2, S3, and A1 wave modes, with the S2 mode being the weakest. Upon implementing the element removal technique in the FEM model (Figure 14b), the S1, S2, S3, and A1 wave modes produce similar results to those of the pipe sample. This agreement between the modified FEM simulation and the experimental data further confirms the effectiveness of the element removal approach in capturing the effects of surface irregularities. In this case, the SSIM and 2D correlation indices increased to 78% and 86%, respectively.

### 5.3. FEM Simulation of Defect Responses

Upon validating the phase velocity maps via experiments conducted on defect-free sections of the pipe, simulations of defect responses were also executed to ascertain the robustness of the model.

**Figure 15:**
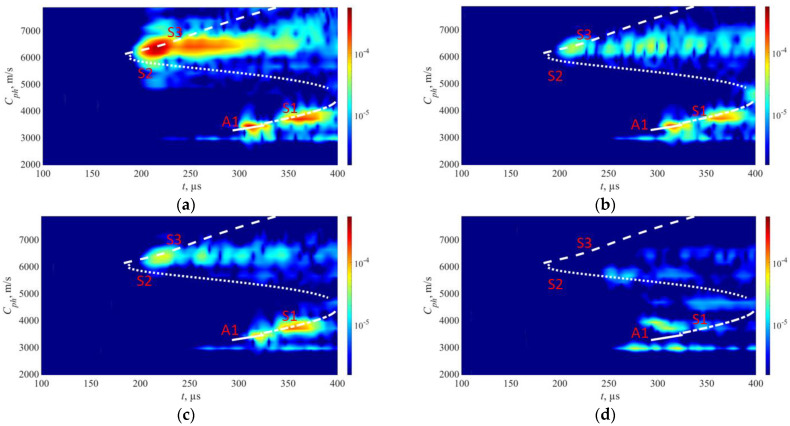
FEM simulation results: (**a**) results from non-defect section; (**b**) localized defect at 30% wall thickness; (**c**) localized defect at 50% wall thickness; (**d**) uniform defect at 50% wall thickness. The color bars are represented in arbitrary units and scaled based on the magnitude of the S3 mode in the defect-free region.

Figure 15 shows the FEM simulated phase velocity maps for defect-free and defected cases that replicate the defects on experimental pipe mock-up.

In the case of the defect-free region presented in Figure 15a, there are notable similarities to the real experiment. The S3, A1, and S1 wave modes are once again clearly visible, with their characteristic phase velocity ranges closely matching those observed in the experiment.

Upon examining a sample in Figure 15b with a localized defect at a depth of 30% in the second case, observed that all modes, including S3, S1, and A1 wave modes, have weakened proportionally, while the S2 mode is not visible. In the case of a localized defect in Figure 15c at a depth of 50%, a comparison with the experimental results reveals that the S3 wave mode has attenuated less compared to the localized defect at depth of 30%, similarly to what was seen in the experiment.

Finally, in the case of a sample in Figure 15d, where there is uniform thinning at a depth of 50% based on FEM simulation results, it is observed that the S3 wave mode experiences the highest level of attenuation. However, the S2 and S1 wave modes remain visible with results comparable to those obtained from the experimental setup. Notably, the presence of the A1 wave mode is absent in this scenario. The substantial attenuation of the S3 mode in the uniform thinning case suggests that the defect may differ from localized defect cases where the S3 mode was detectable.

The SSIM and 2D correlations between FEM simulated maps and experimental maps in cases of localized and uniform defects are presented in Table 1.

### 5.4. Data Arrangement for the VGG-16 Model

As the FEM model was verified both for defect-free and defected cases, a set of FEM models reflecting different stages of the object were simulated. Three distinct cases were examined: no defect (142 models) with varying element removal to simulate different surface conditions, localized defect (135 models), and uniform defect (135 models). The defect sizes ranged from 17% to 80% of the total wall thickness. A total of 412 models were generated for the study. The FEM models were executed on a PC with the following specifications: 64 GB RAM operating at 3400 MHz, an AMD Ryzen 9 5900X CPU (manufactured by AMD, sourced from Santa Clara, CA, USA) with 12 cores running at 4.38 MHz, a Kingston SNV2S2000G solid-state drive (manufactured by Kingston, sourced from Fountain Valley, CA, USA), and an x570 AORUS ELITE motherboard (manufactured by Gigabyte, sourced from New Taipei City, Taiwan). The average runtime for generating a single-phase velocity map, considering one defect scenario, was approximately 168 min, resulting in a total of 1153 calculation hours for the 412 FEM models.

**Figure 16:**
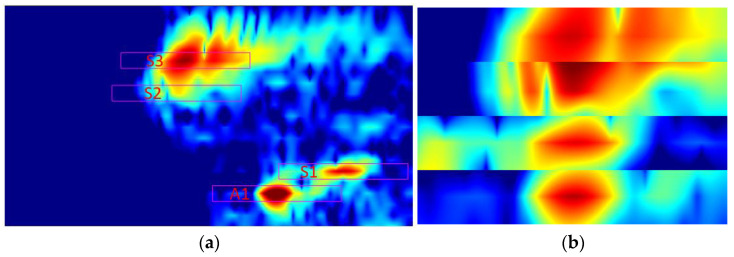
Data preparation for training validation and recognition: (**a**) generated image from new inspection method, where red rectangles indicate selected wave mode zones; (**b**) cropped image with selected wave modes prepared for AI.

Following experimental and FEM validation, the FEM and experimental data were curated in a consistent manner for training and validation. Regions corresponding to the S3, S2, S1, and A1 wave modes from Figure 16a were selectively segmented and collated into a unified image, where each mode zone is locked in a window and normalized using the case with no defect as a reference, as shown in Figure 16b. In doing so, it maintains propagation time and phase velocity information and allows for inspection of the variations of each mode due to the presence of defects. Such approach eliminates areas of pertinent wave modes and reduces data redundancy, yielding to faster and more efficient training of VGG-16 neural network.

**Figure 17:**
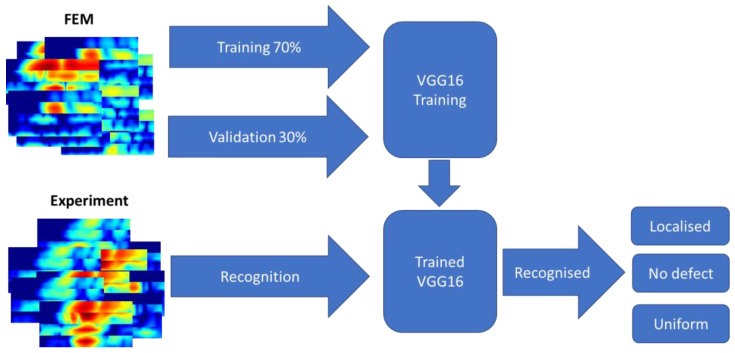
Training validation and recognition setup for VGG16 neural network from FEM and experimental data.

**Figure 18:**
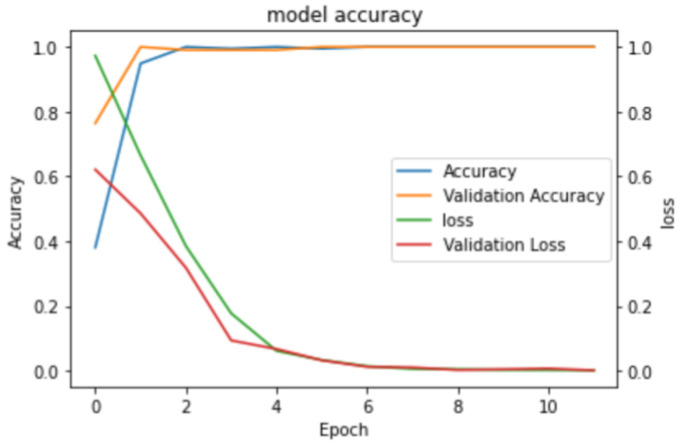
Model training accuracy results.

Out of total 412 FEM models, 70% were used for training and the remaining 30% for validation purposes, enabling us to train the model on the training data and evaluate its performance on unseen testing data. Once the model was trained, it was tested using the experimental data which included samples with 30% (110 experimental results) and 50% localized defects (110 experimental results), a 50% uniform defect (110 experimental results), and defect-free cases (110 experimental results)—in total, there were 440 cases. The experimental measurements were conducted on the pipe mock-up depicted in Figure 1. The data were captured by scanning the tandem of phased arrays along the axial direction of the pipe, specifically targeting the regions around the defects. The training process for the VGG-16 model took a total of 19 min and 21 s. Training setup is visualized in Figure 17, while the accuracy and loss curve are presented in Figure 18. The VGG-16 model was trained for 11 epochs, achieving validation loss of 0.01.

The accuracy results in Figure 18 for the VGG16 neural network model presents a clear progression of improvement over the course of training. At epoch 0, the model had an accuracy of 0.4 and a validation accuracy of 0.75, with a loss of 0.98 and a validation loss of 0.63. By epoch 2, both the accuracy and validation accuracy reached values near 1, while the loss dropped to 0.45 and the validation loss to 0.35. Continuing this trend, at epoch 4, the accuracy and validation accuracy remained near 1, and the loss and validation loss further decreased to approximately 0.1. At epoch 6, the model maintained its near-perfect accuracy and validation accuracy, with both the loss and validation loss values approaching 0.01. The model exhibited good performance on both the training and validation sets, while learning curves showed no significant gap between the training and validation curves, indicating that it was not overfitting. Additionally, to prevent overfitting, stop criteria was used, and model training stopped at the 11th epoch as no further improvements were observed in the subsequent five steps. The VGG-16 model was trained for a total of 19 min until convergence.

**Figure 19:**
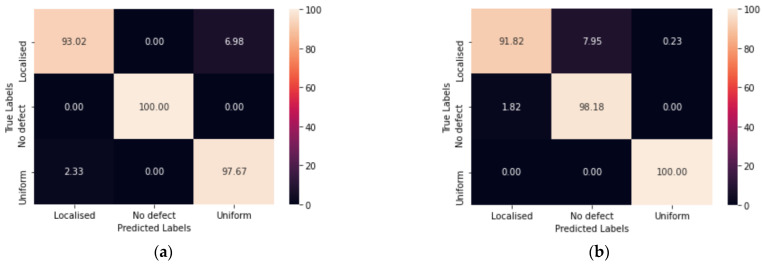
Confusion matrices: (**a**) training validation confusion matrix; (**b**) experimental results.

The confusion matrices of the VGG-16 model are depicted in Figure 19. Figure 19a displays the confusion matrix obtained when 30% of the validation FEM data were utilized. Figure 19b demonstrates the validation of the model using experimental results.

The results in Figure 19a revealed that for localized defects, the model predicted 93.02% accurately, while misclassifying 6.98% as uniform defects. It correctly identified 100% of the no defect cases. In the case of uniform defects, the model achieved a prediction accuracy of 96.67% and misclassified 2.33% as localized defects.

Upon testing the trained network with experimental data in Figure 19b, the model demonstrated the following performance: localized defects were predicted with 91.82% accuracy, 7.95% were misclassified as no defect cases, and 0.23% were identified as uniform defects. For no defect cases, the model achieved an accuracy of 98.18%, with 1.82% misclassified as localized defects. The uniform defect cases had a 100% accuracy.

## 6. Discussion

The results presented in this paper are based on certain limitations. It should be noted that the high accuracy achieved in predicting defect-free cases using synthetic data (Figure 19a) can be partly attributed to the too-slight variations in surface roughness within the datasets. Although different element removal techniques were applied to generate a range of defect-free cases, the resulting models exhibited similar patterns. As a result, a high prediction accuracy for the defect-free class was attained. However, it is important to acknowledge that in real-world scenarios with more complex structures and varying surface roughness, the accuracy of the model may not be as favourable.

The experimental verification demonstrated 100% detection of uniform defects (Figure 19b). However, it should be noted that the experimental mock-up only included one severe uniform defect with a total wall loss of 50%. It is possible that defects with lower wall loss could be mistakenly classified as no defect cases.

Furthermore, our models were trained to identify defects with a minimum wall thickness loss of 17%. It has been observed that smaller changes in wall thickness are often misclassified. Therefore, for reliable classification, a minimum wall thinning of 1.5 mm (in the case of a 9 mm pipe wall) is necessary.

We made a deliberate decision to train the model using synthetic data, which simplifies certain aspects such as neglecting diffraction. However, this approach allows for greater flexibility in expanding training datasets to accommodate new defect parameters. It also enables the model to generalize the problem beyond specific experimental examples and offers the potential to add additional classes or consider different surface conditions, thereby increasing its sophistication.

The current models were unable to predict the exact value of wall thickness loss. However, due to the availability of simulated datasets covering a range of wall thickness losses from 17% to 80%, there is potential to expand the model’s capabilities to predict specific values of wall thinning. This aspect remains a subject of our ongoing and future research.

## 7. Conclusions

In this paper, we proposed a phase velocity mapping method for the detection and classification of wall-thinning defects in pipeline structures. The method is based on selectively exciting different guided wave modes at a constant phase velocity within a specified phase velocity range. By plotting the received responses in a propagation time vs. phase velocity plot, distinct modes propagating in the structure at specific phase velocities can be observed.

In this study, we explored the S1, S2, S3, and A1 modes at an *f* × *d* (frequency–thickness product) of 9 mm × MHz. We found that different modes exhibit unique changes depending on the type and extent of defect present in the structure. For instance, the S3 mode in case of localized defect shows different attenuation, possessing higher amplitude at 50% wall thickness loss. This was found due to the effect of surface roughness, which introduces a variation in the out-of-plane displacements of the S3 mode, resulting in a higher attenuation for small wall thinning. In cases of uniform defects, at 50% of the wall thinning, the S3 mode vanishes as it reaches a cut-off frequency due to the wall loss. The A1 and S1 modes remain present in the case of localized defects, but the phase velocity of the S1 and A1 modes undergo significant change when a uniform defect is present. The absence of the S3 mode and the velocity change of the S1 and A1 modes can serve as an indicator for estimating the thickness and extent of the uniform defect. In cases of localized defects, magnitude variation and velocity change of the S1 mode can be used as a tool for damage identification.

To train the VGG-16 neural network, we used synthetic phase velocity maps as input. These synthetic maps were made realistic by correlating them with measurements from a pipe containing artificial defects. We achieved good agreement between the synthetic maps and the experimental cases, both in defect-free and defected scenarios. This approach allowed us to simulate a wide variety of defected responses at different defect positions and wall thickness losses. Consequently, the maps used for the AI model encompassed a greater range of cases than the experimental mock-up could represent. The VGG-16 model was trained on three classes: defect-free, localized defects, and uniform defects. The assessment of the AI model demonstrated its ability to achieve high predictive accuracy, suggesting its potential suitability for practical applications. When tested with both synthetic and experimental data, the model achieved an average prediction accuracy of 92% for localized defects, 99% for defect-free cases, and 98% for uniform defects. The proposed corrosion detection and classification approach requires further assessment under field conditions, which will be the subject of future research.

In conclusion, our phase velocity mapping method, combined with the AI model, shows promising results for the detection and classification of wall-thinning defects in pipeline structures.

## Figures and Tables

**Table 1:** Comparison between the similarity of experimental and FEM simulated phase velocity maps.

Case	SSIM	CORR2
Localized 30%	82%	89%
Localized 50%	78%	88%
Uniform 50%	91%	95%

## Data Availability

The data generated during this study are currently private due to pending patent applications.
